# Development of a second primary tumor during maintenance immunotherapy in metastatic gingival squamous cell carcinoma: a case report

**DOI:** 10.3389/fonc.2026.1747507

**Published:** 2026-02-27

**Authors:** Xiaoyu Liu, Hanquan Sun, Shangzhong Chen, Shasha He, Min Ouyang, Ping Liu

**Affiliations:** 1 Department of Oncology, Loudi Central Hospital, Loudi, Hunan, China; 2 Department of Oncology, The Second Xiangya Hospital, Central South University, Changsha, Hunan, China; 3 Department of Geriatrics, The Second Xiangya Hospital, Central South University, Changsha, Hunan, China

**Keywords:** case report, gingival squamous cell carcinoma (GSCC), head and neck squamous cell carcinoma (HNSCC), immunotherapy, second primary tumors (SPTs)

## Abstract

Multiple primary tumors are defined as two or more distinct malignancies occurring simultaneously or metachronously in the same patient. This report describes a patient with extensively metastatic gingival squamous cell carcinoma who achieved radiologic complete remission (CR) through combined immunotherapy, chemotherapy, and local radiotherapy. The patient continued immunotherapy maintenance for 38 months. Two years after CR, a second primary tumor emerged. The second tumor was surgically resected, followed by postoperative maintenance therapy with oral Tegafur-Gimeracil-Oteracil (S-1) capsules. As of the last follow-up on January 14, 2025, nearly two years after the second surgery, the patient showed no local recurrence or distant metastasis. This case suggests that while immunotherapy provided excellent overall tumor control, it failed to prevent the occurrence of the second primary tumor. Whether prolonged immunotherapy (38 months vs. the standard 24 months) positively impacts patient prognosis requires further exploration. This case highlights that even after achieving CR with immunotherapy, vigilance for the development of second primary tumors during maintenance therapy is crucial.

## Introduction

Multiple primary tumors are defined as two or more malignant neoplasms occurring simultaneously or metachronously in the same patient. The incidence of multiple primary tumors is approximately 7% among all cancer patients, while the probability in head and neck cancers is about 10.8% (1). Studies have shown that the 5-year overall survival (OS) rate for oral squamous cell carcinoma patients without a second primary tumor is approximately 54%, whereas it is only 23% for those who develop a second primary tumor (2). Therefore, timely diagnosis and treatment of second primary tumors in head and neck cancers are critical. The KEYNOTE-048 trial demonstrated the efficacy and safety of immunotherapy in recurrent or metastatic head and neck squamous cell carcinoma (HNSCC) (3), but the impact of immunotherapy on second primary tumors remains controversial (4, 5). This report describes a patient with widely metastatic gingival squamous cell carcinoma who achieved radiographic complete remission after combined immunotherapy, chemotherapy, and localized radiotherapy. The patient continued immune maintenance therapy for 38 months. Two years after sustained CR, a second primary tumor emerged. Surgical resection and postoperative oral S-1 maintenance therapy were administered. As of the last follow-up on January 14, 2025, nearly two years after the second surgery, the patient showed no local recurrence or distant metastasis. This suggests that while immunotherapy provided excellent overall tumor control, it did not prevent the occurrence of the second primary tumor. Whether the extended duration of immune maintenance therapy (38 months vs. the standard 24 months) positively influences the patient’s prognosis warrants further investigation.

## Case presentation

**Figure 1:**
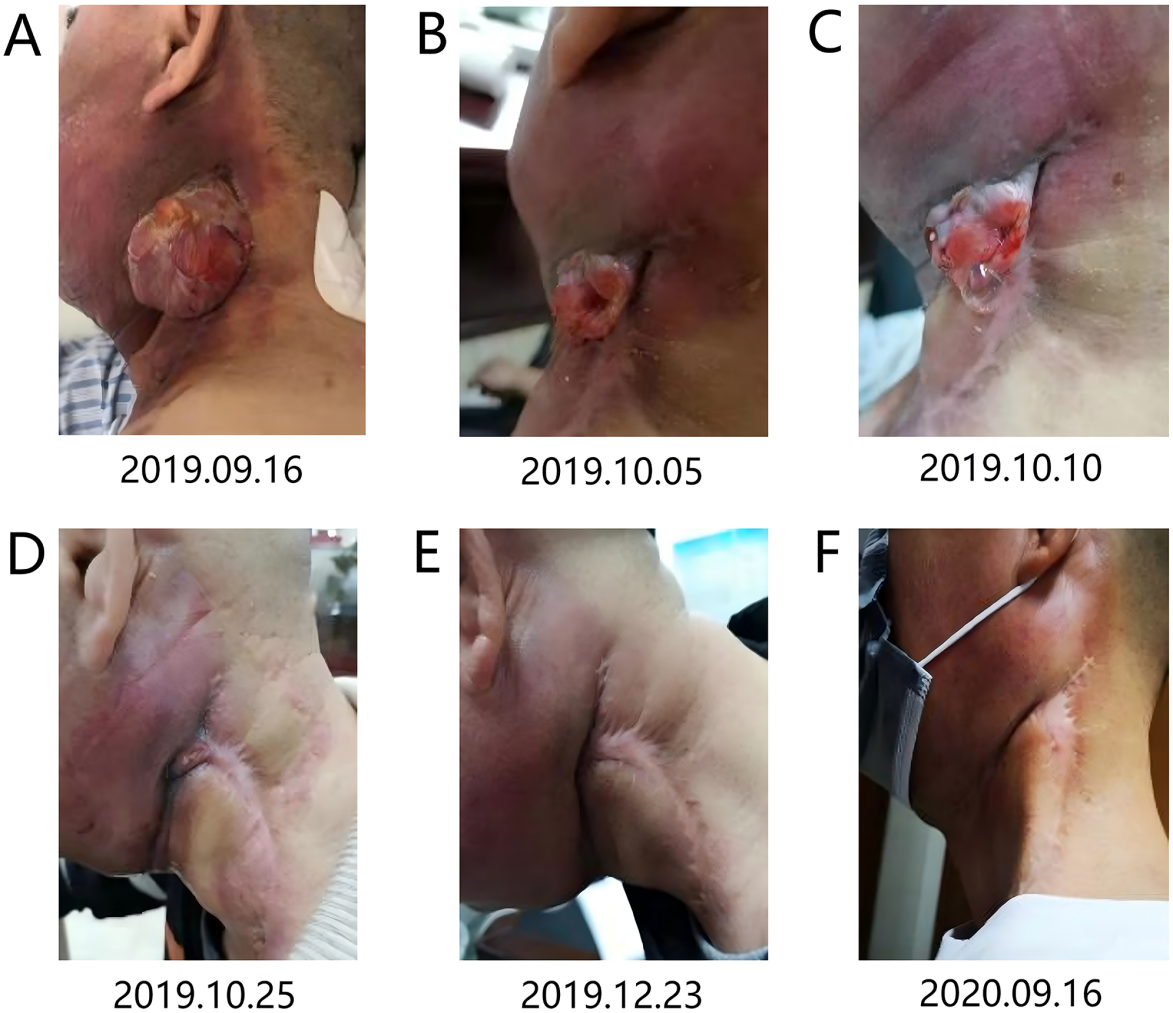
Left neck mass image. **(A)** Taken prior to treatment initiation on September 16, 2019. **(B)** Taken after one cycle of chemotherapy combined with immunotherapy on October 5, 2019. **(C)** Taken after one cycle of chemotherapy combined with immunotherapy on October 10, 2019. **(D)** Taken after two cycles of chemotherapy combined with immunotherapy on October 25, 2019. **(E)** Taken after four cycles of chemotherapy combined with immunotherapy on December 23, 2019. **(F)** Taken after eight cycles of chemotherapy combined with immunotherapy and seven cycles of immunotherapy monotherapy maintenance on September 16, 2020.

**Figure 2:**
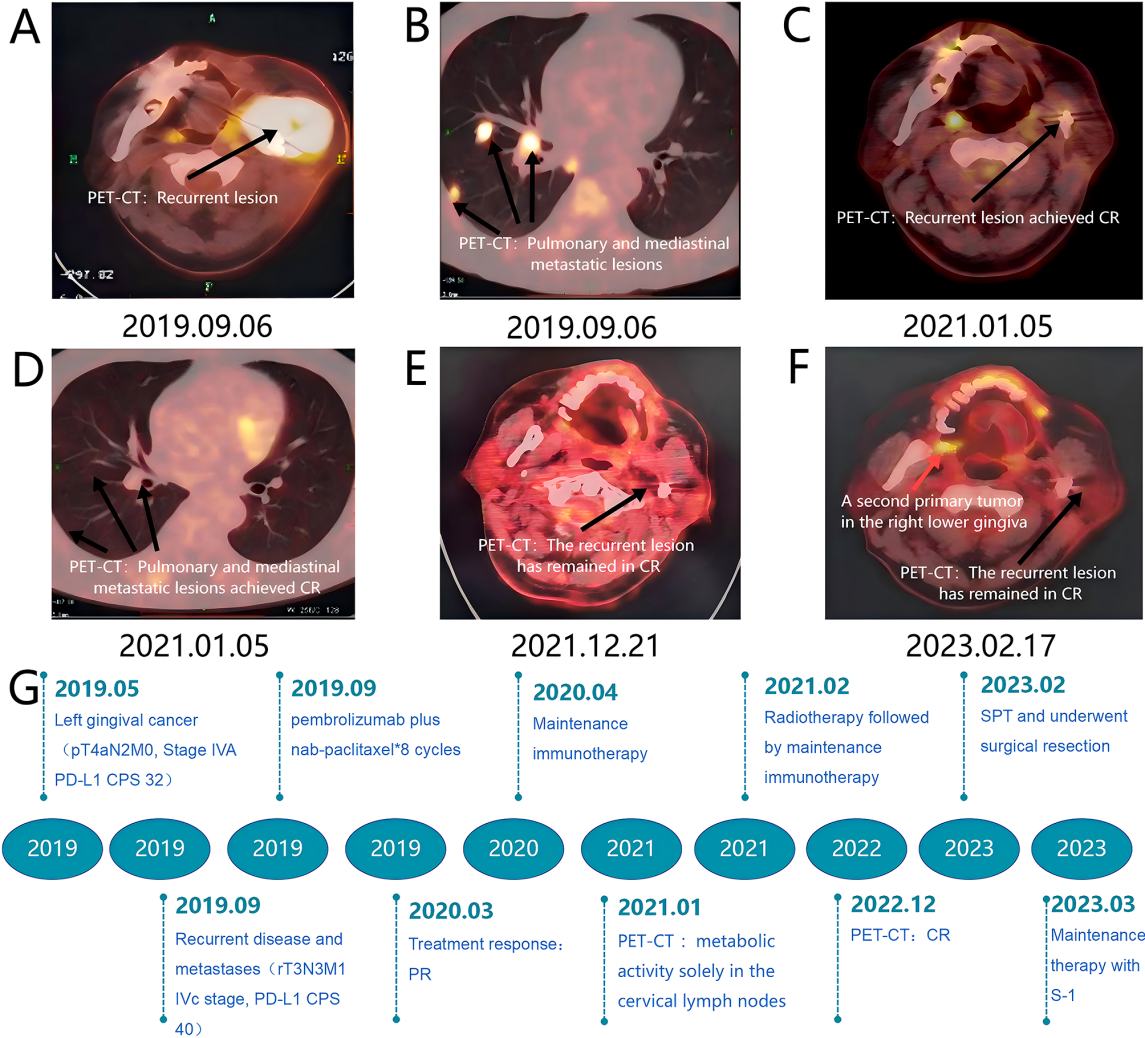
PET-CT images and overview of treatment phase flowchart. **(A)** Image of the primary lesion prior to treatment initiation on September 6, 2019; **(B)** Image of pulmonary and mediastinal metastatic lesions prior to treatment initiation on September 6, 2019; **(C)** Image of the primary lesion achieving partial response (PR) after treatment on January 5, 2021; **(D)** Image of pulmonary and mediastinal metastatic lesions achieving complete response (CR) after treatment on January 5, 2021; **(E)** Image of the primary lesion achieving complete response (CR) after treatment on December 21, 2021; **(F)** Image of the second primary tumor lesion on February 17, 2023. **(G)** Overview of treatment phase flowchart.

A 48-year-old male with a 20-pack-year smoking history (1 pack/day) and over 20 years of betel nut chewing history, but no history of alcohol abuse, presented with progressive pain in the left posterior lower gingiva in March 2019. Biopsy at the Second Xiangya Hospital of Central South University revealed focal carcinoma, leading to radical surgery on May 20, 2019 (left gingivo-mandibular-neck composite resection + left fibular myocutaneous flap transfer and reconstruction + tracheostomy). Postoperative pathology confirmed moderately differentiated squamous cell carcinoma, staged as pT4aN2bM0 IVA stage (tumor invading jawbone, ipsilateral level I and II lymph node metastases, AJCC 8th edition). No adjuvant therapy was administered initially. In August 2019, a left neck mass was detected, which gradually increased in size. In early September 2019, the patient presented to the Oncology Department of the Second Xiangya Hospital with a mass measuring approximately 7×8 cm (**Figure 1A**). PET-CT revealed hypermetabolic lesions in the left surgical area, lungs, and mediastinal lymph nodes (**Figures 2A, B**), consistent with recurrent gingival carcinoma with lung and mediastinal lymph node metastases. The diagnosis was gingival squamous cell carcinoma (rT3N3M1 IVc stage, PD-L1 CPS score 40). Following multidisciplinary team (MDT) discussion, therapy with pembrolizumab 200mg + nab-paclitaxel 400mg was initiated for eight cycles (21-day intervals), followed by pembrolizumab maintenance. After the first cycle, partial regression of the neck mass was observed (**Figures 1B, C**), with near-complete resolution by the second cycle (**Figure 1D**) and complete resolution by the fourth cycle (**Figure 1E**). By September 2020, after seven cycles of pembrolizumab monotherapy, only a surgical scar remained (**Figure 1F**). A January 2021 PET-CT showed complete response (CR) in lung and mediastinal metastases, with partial response (PR) at the primary site (**Figures 2C, D**). A second MDT recommended radiotherapy targeting the primary site (PGTV: 70Gy/2.25Gy/31F, PTV: 60Gy/1.94Gy/31F, Monday to Friday, covering the primary site and bilateral neck levels I-V lymph nodes) alongside continued pembrolizumab maintenance. Efficacy was maintained as PR during this period. A December 2021 PET-CT confirmed systemic CR (**Figure 2E**). Immunotherapy maintenance continued until February 2023, when the patient reported discomfort in the right lower gingiva for over two months. PET-CT revealed a hypermetabolic lesion in the right buccal-palatal and mandibular alveolar regions (**Figure 2F**), without recurrence at the primary site. A third MDT recommended surgical resection in March 2023. Postoperative pathology confirmed moderately to poorly differentiated squamous cell carcinoma. A fourth MDT consensus classified this as a second primary tumor. Postoperatively, oral S-1 maintenance therapy was initiated (60mg twice daily, 28 days on/14 days off per cycle). Follow-up imaging in May 2023, July 2023, November 2023, May 2024, and January 2025 (CT, MRI) showed no recurrence. The patient remains stable on S-1 therapy, in good general condition. The patient’s ECOG PS score throughout the treatment period was 1.

## Patient perspective, quality of life, and psychological aspects

Throughout the treatment course, the patient reported significant alterations in quality of life and psychological well-being. Following the initial radical surgery, he experienced a noticeable decline in speech clarity and mild impairment of masticatory function, which impacted his dietary choices and social interactions, contributing to reduced self-confidence due to visible facial changes. Upon diagnosis of recurrence with distant metastasis, he expressed profound anxiety and pessimism regarding his prognosis. However, the remarkable response to immunochemotherapy, leading to sustained disease control, markedly improved his outlook. During the extended period of maintenance therapy, he valued his regained health above all else and maintained a positive and cooperative attitude. The second extensive surgery resulted in further impairment of speech and a more significant reduction in chewing efficiency. Despite these challenges, he retains the ability for oral intake with adapted food consistency and has demonstrated remarkable resilience. He continues to express strong hope and adherence to the ongoing maintenance therapy with oral S-1, focusing on functional adaptation and long-term survival.

## Discussion

**Figure 3:**
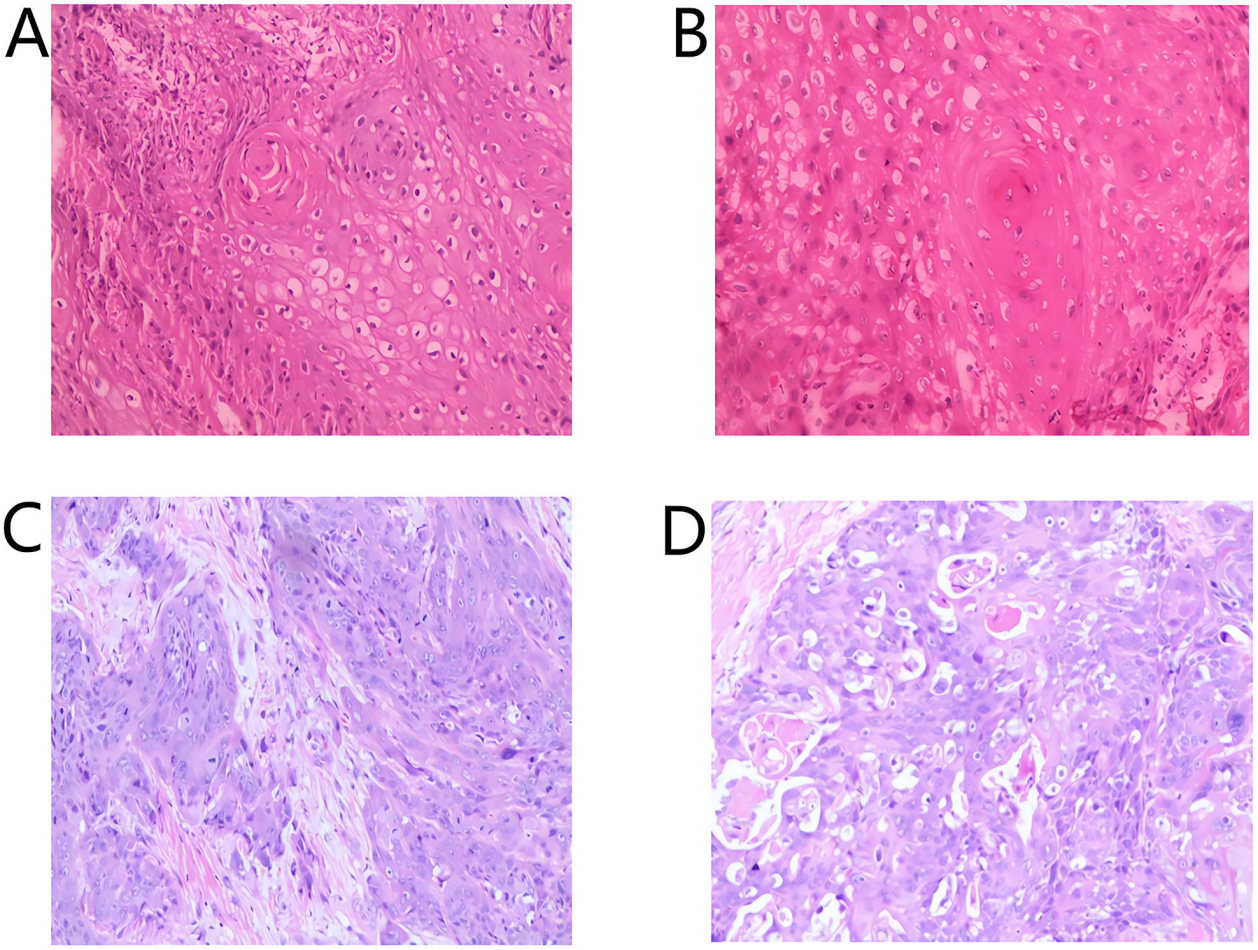
Histopathological images. **(A, B)** Present histopathological images from May 2019 (HE staining, 40×); **(C, D)** show corresponding images from March 2023 (HE staining, 40×).

Second primary tumors must meet three criteria: (1) both tumors must be malignant, (2) anatomically distinct, and (3) metastasis from the first tumor must be excluded (6). In clinical practice, the last criterion requires detailed analysis. In this case, the first primary tumor originated in the left posterior lower gingiva, while the second arose in the right buccal-palatal and mandibular alveolar regions. Both were histologically malignant and anatomically separate, fulfilling the first two criteria. Differentiating metastasis from a second primary tumor was challenging due to shared squamous cell origin and overlapping histopathology. Regarding the timing of second primary tumor occurrence, one study reported a median interval of 53.6 months for second primary tumors appearing in the head and neck region (7). In this case, the interval was 46 months (May 2019 to March 2023), close to the average. Therapeutically, the patient achieved CR after immunotherapy combined with chemotherapy and radiotherapy. The emergence of the second tumor during ongoing maintenance therapy, while the primary site and lungs remained controlled, suggests differential drug sensitivity between the two tumors. The combined positive score (CPS) was 32 for the first tumor versus 10 for the second. Histologically, the first was moderately differentiated, while the second was moderately-to-poorly differentiated (**Figure 3**). The patient’s smoking history, betel nut chewing, and prior radiotherapy are established risk factors for second primaries (2, 8, 9). Furthermore, oral cancers rarely metastasize to contralateral sites, supporting the diagnosis of a second primary.

Analyzing the potential mechanisms for the development of the second primary tumor during the 38 months of immunotherapy, the primary mechanism is closely related to Cancer Immunoediting. The selective pressure exerted by treatment eliminates highly immunogenic tumor cell clones but may simultaneously select for and promote the survival and expansion of low immunogenicity clones capable of immune escape. These cells evade immune surveillance through mechanisms such as loss of MHC class I molecules, defects in antigen presentation machinery, or mutations in interferon signaling pathways. Additionally, high intratumoral heterogeneity (ITH) means treatment cannot eradicate all subclones; residual drug-resistant cells may serve as the origin for the second primary tumor. Concurrently, certain immunotherapies might temporarily affect the body’s overall immune surveillance function, impairing its ability to effectively identify and eliminate nascent or pre-existing micro-lesions, ultimately leading to the occurrence of the second primary tumor (10, 11). Furthermore, the region where the second primary tumor emerged received partial low-dose irradiation during the patient’s radiotherapy. Studies suggest that low-dose radiation may promote the development of second primary tumors (12). Additionally, the patient’s decades-long betel nut chewing habit is significant. Previous research indicates that long-term exposure of oral mucosa to betel nut significantly increases the incidence of oral precancerous lesions among chewers, with approximately 1%-18% of these lesions progressing to malignancy over several years (13–16). The patient’s smoking history (20 pack-years) is also relevant; studies found that smoking more than 20 cigarettes per day is associated with an increased risk of second primary cancer among head and neck cancer survivors compared to non-smokers (17).

Given the high propensity for second primary tumors in head and neck cancer survivors and their poor prognosis, patients achieving CR after aggressive treatment require heightened vigilance for the possibility of second tumors. Close follow-up and evaluation of common sites like the lungs and oral cavity are essential (18). Furthermore, the impact of immunotherapy on second primary tumors remains debated (4, 5, 19). In this case, a second primary tumor emerged during maintenance therapy after achieving CR, indicating the need for more research to clarify the optimal duration of immunotherapy maintenance and its effect on second primary tumors. This patient had a CPS of 32, categorizing him as a favorable candidate for immunotherapy per KEYNOTE-048. He presented with postoperative recurrence and multiple lung metastases. After 8 cycles of pembrolizumab plus chemotherapy, distant metastases achieved CR and the primary site showed PR. Subsequent radiotherapy and continued immunotherapy maintenance led to CR at the primary site, indicating excellent response of the first primary tumor to the combined regimen. The second primary tumor emerged one year after achieving CR during maintenance therapy. It was managed surgically followed by S-1 maintenance, and the patient has remained recurrence-free for nearly two years post-second surgery. The immunotherapy duration (38 months) far exceeded the guideline-recommended 24 months and was associated with favorable outcomes. However, a retrospective study suggested no overall survival advantage for patients continuing immunotherapy beyond 24 months compared to those stopping at 24 months (20). Whether prolonged immunotherapy beyond the standard duration benefits prognosis requires further investigation. The extended immunotherapy duration and overall treatment strategy in this case offer valuable insights for clinical practice. This case also invites reflection on the broader management strategy for giant, recurrent, or metastatic head and neck tumors. Traditionally, extensive salvage surgery has been a cornerstone for locally advanced disease. Immunotherapy is increasingly challenging the primacy of aggressive surgery in selected cases. This shift is exemplified in the management of giant carcinomas, such as carcinoma ex-pleomorphic adenoma (CXPA) of the parotid gland. As discussed by Bratiloveanu et al. (2024), giant CXPA with skin invasion presents a major therapeutic challenge, often pushing management towards extensive salvage surgery (21). However, they highlight that immunotherapy has shown promising activity in similar advanced salivary gland carcinomas (22, 23). In the clinical decision-making process to differentiate a second primary carcinoma from a metastasis, our institution faced certain diagnostic limitations. Although we made a comprehensive judgment based on clinicopathological features (such as the contralateral location and differing degrees of differentiation) and a long disease-free interval, the most conclusive molecular evidence was unavailable.As this is a single case report, it cannot conclude that extending immunotherapy maintenance improves clinical outcomes, nor can it definitively establish whether immunotherapy promotes the development of second primary tumors.

## Data Availability

The raw data supporting the conclusions of this article will be made available by the authors, without undue reservation.

## References

[1] Wang X Zeng M Ju X Lin A Zhou C Shen J . Correlation between second and first primary cancer: systematic review and meta-analysis of 9 million cancer patients. Br J Surg. (2024) 111:znae044. doi: 10.1093/bjs/znad377, PMID: 38055899

[2] Ko HH Cheng SL Lee JJ Chen HM Wang CW Cheng SJ . Factors influencing the incidence and prognosis of second primary tumors in patients with oral squamous cell carcinoma. Head Neck. (2016) 38:1459–66. doi: 10.1002/hed.24457, PMID: 27061604

[3] Burtness B Harrington KJ Greil R Soulieres D Tahara M de Castro G Jr . Pembrolizumab alone or with chemotherapy versus cetuximab with chemotherapy for recurrent or metastatic squamous cell carcinoma of the head and neck (KEYNOTE-048): a randomised, open-label, phase 3 study. Lancet. (2019) 394:1915–28. doi: 10.1016/S0140-6736(19)32591-7, PMID: 31679945

[4] Heudel P Chabaud S Perol D Ray-Coquard I Blay JY . Reduced risk of second primary cancer in patients treated with immune checkpoint inhibitors for a first cancer. Ann Oncol. (2020) 31:1773–5. doi: 10.1016/j.annonc.2020.09.001, PMID: 32916266

[5] Deng W Wang Y Liu X Liu J Wang L Yang Z . Assessment of trends in second primary cancers in patients with metastatic melanoma from 2005 to 2016. JAMA Netw Open. (2020) 3:e2028627. doi: 10.1001/jamanetworkopen.2020.28627, PMID: 33295975 PMC7726633

[6] Warren SJG . Multiple primary Malignant tumors: a survey of the literature and a statistical study. Am J Cancer. (1932) 93:779.

[7] Herranz Gonzalez-Botas J Varela Vazquez P Vazquez Barro C . Second primary tumours in head and neck cancer. Acta Otorrinolaringol Esp. (2016) 67:123–9. doi: 10.1016/j.otorri.2015.04.001, PMID: 26386656

[8] Dracham CB Shankar A Madan R . Radiation induced secondary Malignancies: a review article. Radiat Oncol J. (2018) 36:85–94. doi: 10.3857/roj.2018.00290, PMID: 29983028 PMC6074073

[9] Chuang SC Scelo G Tonita JM Tamaro S Jonasson JG Kliewer EV . Risk of second primary cancer among patients with head and neck cancers: A pooled analysis of 13 cancer registries. Int J Cancer. (2008) 123:2390–6. doi: 10.1002/ijc.23798, PMID: 18729183

[10] Roerden M Spranger S . Cancer immune evasion, immunoediting and intratumour heterogeneity. Nat Rev Immunol. (2025) 25:353–69. doi: 10.1038/s41577-024-01111-8, PMID: 39748116

[11] O’Donnell JS Teng MWL Smyth MJ . Cancer immunoediting and resistance to T cell-based immunotherapy. Nat Rev Clin Oncol. (2019) 16:151–67. doi: 10.1038/s41571-018-0142-8, PMID: 30523282

[12] Eidemuller M Holmberg E Jacob P Lundell M Karlsson P . Breast cancer risk and possible mechanisms of radiation-induced genomic instability in the Swedish hemangioma cohort after reanalyzed dosimetry. Mutat Res. (2015) 775:1–9. doi: 10.1016/j.mrfmmm.2015.03.002, PMID: 25839758

[13] Sharan RN Mehrotra R Choudhury Y Asotra K . Association of betel nut with carcinogenesis: revisit with a clinical perspective. PloS One. (2012) 7:e42759. doi: 10.1371/journal.pone.0042759, PMID: 22912735 PMC3418282

[14] Chen YJ Chang JT Liao CT Wang HM Yen TC Chiu CC . Head and neck cancer in the betel quid chewing area: recent advances in molecular carcinogenesis. Cancer Sci. (2008) 99:1507–14. doi: 10.1111/j.1349-7006.2008.00863.x, PMID: 18754860 PMC11159516

[15] Yang YH Chen CH Chang JS Lin CC Cheng TC Shieh TY . Incidence rates of oral cancer and oral pre-cancerous lesions in a 6-year follow-up study of a Taiwanese aboriginal community. J Oral Pathol Med. (2005) 34:596–601. doi: 10.1111/j.1600-0714.2005.00266.x, PMID: 16202079

[16] Warnakulasuriya S Trivedy C Peters TJ . Areca nut use: an independent risk factor for oral cancer. BMJ. (2002) 324:799–800. doi: 10.1136/bmj.324.7341.799, PMID: 11934759 PMC1122751

[17] Shiels MS Gibson T Sampson J Albanes D Andreotti G Beane Freeman L . Cigarette smoking prior to first cancer and risk of second smoking-associated cancers among survivors of bladder, kidney, head and neck, and stage I lung cancers. J Clin Oncol. (2014) 32:3989–95. doi: 10.1200/JCO.2014.56.8220, PMID: 25385740 PMC4251962

[18] Jones AS Morar P Phillips DE Field JK Husband D Helliwell TR . Second primary tumors in patients with head and neck squamous cell carcinoma. Cancer. (1995) 75:1343–53. doi: 10.1002/1097-0142(19950315)75:6<1343::AID-CNCR2820750617>3.0.CO;2-T 7882285

[19] Heudel P Chabaud S Perol D Flechon A Fayette J Combemale P . Immune checkpoint inhibitor treatment of a first cancer is associated with a decreased incidence of second primary cancer. ESMO Open. (2021) 6:100044. doi: 10.1016/j.esmoop.2020.100044, PMID: 33516148 PMC7844579

[20] Sun L Bleiberg B Hwang WT Marmarelis ME Langer CJ Singh A . Association between duration of immunotherapy and overall survival in advanced non-small cell lung cancer. JAMA Oncol. (2023) 9:1075–82. doi: 10.1001/jamaoncol.2023.1891, PMID: 37270700 PMC10240399

[21] Bratiloveanu M Dumitru M Marinescu AN Serboiu C Patrascu OM Costache A . Challenges in the management of giant carcinoma ex-pleiomorphic adenoma of the parotid gland in a single tertiary center. Med (Kaunas). (2024) 61:37. doi: 10.3390/medicina61010037, PMID: 39859019 PMC11766963

[22] Bugia L Jungbauer F Zaubitzer L Horner C Merx K Yasser AM . Nivolumab as a promising treatment option for metastatic salivary duct carcinoma. J Immunother. (2024) 47:258–62. doi: 10.1097/CJI.0000000000000513, PMID: 38545827 PMC11299898

[23] Yu W Ding C Li K . Partial response to RC-48 as palliative treatment in a patient with locallocally advanced carcinoma ex pleomorphic adenoma harboring a HER2 mutation: a case report. Oral Oncol. (2023) 146:106541. doi: 10.1016/j.oraloncology.2023.106541, PMID: 37595449

